# Adaptation, fitness landscape learning and fast evolution

**DOI:** 10.12688/f1000research.18575.2

**Published:** 2019-09-13

**Authors:** John Reinitz, Sergey Vakulenko, Dmitri Grigoriev, Andreas Weber

**Affiliations:** 1 Departments of Statistics, Ecology and Evolution, Molecular Genetics and Cell Biology, University of Chicago, Chicago, IL, USA; 2 Saint Petersburg National Research University of Information Technologies, Mechanics and Optics, Saint Petersburg, Russian Federation; 3 CNRS, Mathématiques, Université de Lille, Villeneuve d'Ascq, France; 4 Department of Computer Science, University of Bonn, Bonn, Germany

**Keywords:** evolution, gene networks, fitness landscape learning

## Abstract

We consider evolution of a large population, where fitness of each organism is defined by many phenotypical traits. These traits result from expression of many genes. Under some assumptions on  fitness we prove that such model organisms  are capable, to some extent, to recognize the fitness landscape. That fitness landscape learning sharply reduces the number of mutations needed for adaptation. Moreover, this learning increases phenotype robustness with respect to mutations, i.e., canalizes the phenotype.  We show that learning and canalization work only when evolution is gradual. Organisms can be adapted to  many constraints associated with a hard environment, if that environment becomes harder step by step. Our results explain why evolution can involve genetic changes of a relatively large effect and why the total number of changes are surprisingly small.

## 1 Introduction

A central idea of modern biology is that evolution proceeds by mutation and selection. This process may be represented as a walk in a fitness landscape leading to fitness increase and slow adaptation ^1^. According to classical ideas this walk can be considered a sequence of small random steps with small phenotypic effects. Nevertheless, there is a limited amount of experimental support for this idea ^2^ and some experimental evidence that evolution can involve genetic changes of a relatively large effect and that the total number of changes are surprisingly small ^3^. Another intriguing fact is that organisms are capable of making adaptive predictions of environmental changes ^4^.

To explain those facts new evolutionary concepts have been suggested (see the review by 5 and references therein). The main idea is that a population can “learn” (recognize) fitness landscapes ^5– 7^. The approach developed in these works is a generalization of ideas from machine learning in which learning (regression to data) is viewed as selection and generalization (interpolation or extrapolation) is viewed as adaptation.

A mathematical basis for investigation of evolution learning problems has been developed by 8. However, this work uses a simplified model, where organisms are represented as Boolean circuits seeking an “ideal answer” to environmental challenges. These circuits involve *N _g_* Boolean variables that can be interpreted as genes, and the ideal circuit answer maximizes the fitness. A similar model was studied numerically by 7 to confirm the theory of “facilitated variation” explaining the appearance of genetic variations which can lead to large phenotypic ones. In the work by 9 a theory of the evolution of these Boolean circuits was advanced. It was shown that, under some conditions—weak selection, see 10—a polynomially large population over polynomially many generations (polynomial in *N _g_*) will end up almost surely consisting exclusively of assignments, which satisfy all constraints. This theorem can shed light on the problem of the evolution of complex adaptations since that satisfiability problem can be considered as a rough mathematical model of adaptation to many constraints.

In 6 it is shown that, in the regime of weak selection, population evolution can be described by the multiplicative weight update algorithm (MWUA), which is a powerful tool, well known in theoretical computer science and a generalization of such famous algorithms as Adaboost and others ^11^. Note that in 6 infinitely large populations are investigated whereas the results of 9 hold only for finite populations and take into account genetic drift.

Evolution Computation(EC) problems are considered recently by many papers ^12– 16^ mainly for artificial test fitness functions like OneMax or LeadingOnes (for an overwiew of EC problems, see 17).

In this paper, we investigate adaptation and the fitness landscape learning problem for more realistic fitness function This fitness can model adaptation for insects and connected with a fundamental hard combinatorial problem: *K*-SAT.

The main results can be outlined as follows. We show that, in a fixed environment, genes can serve as learners in the machine learning sense. Indeed, if an organism has survived for a long period, this fact alone constitutes important information, which can be used. The biological interpretation of this fact is simple: if a population is large enough and mutations are sufficiently rare, deleterious mutations are eliminated by purifying selection. Hence, those non-neutral mutant alleles which have become fixed in natural populations will, with probability close to 1, be adaptive and cause a positive increment of fitness (see Theorem 3.1 and Theorem 3.2 in Subsection 3.1 and Subsection 3.2). We obtain mathematical results, which allows us to estimate the reduction of mutation number due to that learning landscape procedure. Learning can sharply reduce the number of mutations needed to form a phenotypic trait useful for adaptation that is consistent with experimental data mentioned above (see 3).

Another important result is as follows. We estimate the accuracy of fundamental Nagylaki equations ^6, 10^ for a realistic population model, where the population size is bounded and a non-zero mutation rate is taken into account (in the case of asexual reproduction). Those accuracy estimates are fulfilled for all possible values of mutation rates and population sizes.

## 2 Model

In this section, we describe our model and mathematical approach.

### 2.1 Genome

We assume that the genotype can be described by Boolean strings of length *N _g_*, where *N _g_* is the number of genes. Then

$$s=(s_{1},s_{2},\ldots ,s_{N_{g}}),\;s_{i}\in S=\left\{0,1\right\},\;s\in S^{N_{g}},\;(2.1)$$

where *s _i_* = 1 means that gene *i* is activated (switched on) and *s _i_* = 0 means that it is repressed (switched off). Correspondence between Boolean hypercube and genotypes is considered for example in 12.

### 2.2 Phenotypic traits

Although phenotype is controlled by genes, it is also influenced by environmental conditions and various epigenetic processes. In this paper, we suppose that phenotypic traits are controlled by genotype only. We consider levels *f _j_* of expressions of those traits as real variables in the interval (0, 1). Then the vector *f* = ( *f*_1_, . . . , *f _N _b__*) can be considered to represent the organismal phenotype. We suppose that

$$f_{j}=f_{j}(s),\;j=1,\ldots ,N_{b},\;(2.2)$$

where *f _j_* ∈ (0, 1) is a real valued function of the Boolean string *s*, the genotype.

Only a part of *s _i_* is involved in *f _j_*. Namely, for each *j* we have a set of indices *K _j_* = { *i*_1_, *i*_2_, . . ., *i _n _j__*} such that *f _j_* depends on *s _i_* with *i* ∈ *K _j_*, so that

$$f_{j}(s)=f_{j}(s_{i_{1}},s_{i_{2}},\ldots ,s_{i_{n_{j}}}),$$

where *i _l_* ∈ *K _j_* and *n _j_* is the number of genes involved in the control of the trait expression.

The representation of phenotype by the quantities *f _j_* is suggestive of quantitative traits because the *f _j_* are real valued. The limiting values of 0 or 1 suggest another interpretation, however, in terms of cell type. Multicellular organisms consist of cells of different types. One can suppose that the organismal phenotype is defined completely by the corresponding cell pattern. The cell type *j* is determined by morphogenes, which can be identified as gene products or signaling molecules that can change cell type (or genes that code for signaling molecules that can determine cell types or cell-cell interactions and then finally the cell pattern). The morphogene activity is defined by ( 2.2).

We further suppose:

**Assumption M**. *Assume activities f _j_ have the following properties.*

*The sets K _j_ are independent uniformly random subsets of S _g_* = {1, . . . *N _g_*}

$$K_{j}=\left\{i_{1},\ldots ,i_{n_{j}}\right\},\;i_{l}\in S_{g},\;l=1,\ldots ,n_{j}.\;(2.3)$$

We denote the total number of genes involved in regulation of all *f _j_* by *N _r_*, where $N_{r}=\sum n_{j}\leq KN_{b}.$

Assumption **M** implies that the genetic control of the phenotype is organized, in a sense, randomly, and that only a portion of the full set of genes controls phenotypic traits. That modularity of gene control is well known from experimental data (see 18, 19) and for evolution computation problems it was studied, for example, in 16.

Consider an example, where the assumption **M** holds, where we have a saturated expression, inspired by earlier work ^20, 21^. Let

$$f_{j}=\sigma (\sum_{i=1}^{N_{g}}w_{ji}s_{i}-h_{j}),\;(2.4)$$

where *j* = 1, . . . , *N _b_*. Here *σ*( *z*) is a sigmoidal function of real *z* such that

$$\sigma (+\infty )=1,\;\sigma (-\infty )=0,\;\sigma^{\prime }(z)>0\;\forall z\;(2.5)$$

and *w _ij_*, *h _j_* are some coefficients (their meaning will be explained below). As an example, we can take *σ*( *S*) = (1 + exp( *−bS*)) ^−1^, where *b >* 0 is a sharpness parameter. Note that for large *b* this sigmoidal function tends to the step function and for *b* = + *∞* our model becomes a Boolean one. The parameters *h _j_* defines thresholds for trait expression ^20^. The relation ( 2.4) can be interpreted as a simple mathematical model for quantitative trait locus (QTL) action.

To understand the role of *h _j_* consider a trait *f _j_* and suppose that for a well adapted organism *f _j_ ≈* 1. Let, for simplicity, *w _ij_* take the values 1, 0, or *−*1. Then the parameter *h _j_* defines how many genes involved in the control of the *f _j_* expression should be activators and how many should be repressors. Let the numbers of activator and repressor genes be $n_{j}^{\pm }$, respectively.

Then *f _j_ ≈* 1 if $n_{j}^{+}-n_{j}^{-}\gg h_{j}.$

One can suppose that *h _j_* describes a direct influence of environment on phenotype, such as stress, that can exert epigenetic effects. In Section 2.6 using data from 22 we will show that the model defined by ( 2.4) are capable to describe main topological characteristics of really observed fitness functions in the case of mimicry, camouflage and thermoregulation for insects.

Let us introduce the matrix **W** of size *N _b_* × *N _g_* with the entries *w _ij_*. The coefficients *w _ji_* determine the effects of terminal differentiation genes (see 23), and hence encodes the genotype-phenotype map. We assume that the coefficients *w _ji_* are random, with the probability that *w _ji_* > 0 or that *w _ji_* < 0 is *β/*2 *N*, where *β* > 0 is a parameter. This quantity *β* << *N* defines a genetic redundancy, i.e., averaged numbers of genes that control a trait. Note that then large *β* ≫ 1 one has *n _j_* < 4 *β* with the probability Pr *_β_*, which is exponentially close to 1: Pr _*β*_ > 1 – exp(−0.1 *β*), thus, the number *n _j_* are bounded.

### 2.3 Fitness

We know little about the details of how fitness relates to the phenotype of multicellular organisms, and for that reason classic neo-Darwinian theory takes fitness to be a function of genotype. Some models which take account of epistasis have been proposed ^24^. The random field models assign fitness values to genotypes independently from a fixed probability distribution. They are close to mutation selection models introduced by 25, and can be named House of Cards (HoC) model. The best known model of this kind is the NK model introduced by Kauffman and Weinberger ^26^, where each locus interacts with *K* other loci. Rough Mount Fuji (RMF) models are obtained by combining a random HoC landscape with an additive landscape models ^27^. In evolution computations (EC) some artificial fitness models were used, for example OneMax and Leading Ones to test evolution algorithms, see for example ^15^.

In this work, we use the classical approach of R. Fisher by introducing an explicit representation of phenotype, *f*, and allow it to determine fitness through interaction with an environment *b*. That is, we assume that the phenotype is completely determined by the phenotype trait expression, and thus the fitness depends on the genotype *s* via *f _j_*.

We express the relative fitness *F* and its dependence on environment *b* via an auxiliary function *W* via the relation

$$F(s,b)=K_{F}\exp(W(s,b)),\;(2.6)$$

where *K _F_* is a positive constant and *b* = ( *b*_1_,..., *b _N _b__*) is a vector consisting of coefficients *b _j_*, respectively. Below we refer to *W* as a fitness potential, and we assume that

$$W(s,b)=\sum_{j=1}^{N_{b}}b_{j}f_{j}(s).\;(2.7)$$

Sometimes, if the parameter *b* is fixed, we shall omit the corresponding argument in notation for *W* and *F*.

We consider fitness as a numerical measure of interactions between the phenotype and an environment. For a fixed environment, this idea gives us the fitness of classical population genetics. A part of the fitness, however, depends on the organism developing properly and for now we represent it as independent of the environment, although we are aware that this is not always the case. Note that some coefficients *b _j_* may be negative and others may be positive, and that the model ( 2.7) can describe gene epistatic effects via dependence of *f _j_* on *s* if *f _j_* are nonlinear in *s*.

The expression ( 2.7) can serve as a rough approximation of the fitness function in the case of insects such as grasshoppers or fruit flies. In fact, important factors, which determine insect survival, are thermoregulation, mimicry and camouflage levels ^18, 22, 28^. All those factors depend on colour pigmentation pattern. Blackwhite pigmentation patterns can be roughly described by vectors *f* = ( *f*_1_, *f*_2_,..., *f _N _b__*), where *f _j_ ≈* 1 and *f _j_ ≈* 0 mean that the cell *j* is black, or white, respectively, Then thermoregulation depends on $\sum_{j}f_{j}.$ The mimicry level can be approximately defined by expression $\sum_{j}\left|f_{j}-f_{j}^{*}\right|$, where f_*_ is a target pattern corresponding to an insect to mimic. Colour patterns can be also described by classical RGB formalism.

The representation of the fitness as a sum of terms ( 2.7) is of course a rough approximation; however if assumption **M** holds that representation is consistent with important observed facts. First, mutations have been identified that alter one part of the pigment pattern without affecting any other. This independence of different pattern parts can be explained by the modular organization of the genetic regulation that controls pigmentation. In the course of evolution, different aspects of the pigment pattern have clearly evolved independently of each other ^18^. Second, the topology of the fitness landscapes was studied in 22 by field experiments in the case of insect mimicry. Main conclusions are as follows. A number of studies of fitness landscapes in natural populations have demonstrated low fitness of intermediate phenotypes, i.e., existence of valley in the fitness landscape. It is found ^22^ that natural selection promotes genetic architecture preventing the expression of intermediate phenotypes. Close fitness peaks are separated by ridges, favouring colour pattern switches and allowing drift from local peaks.

In Section 2.6 we will show that the fitness model defined by ( 2.4) and ( 2.7) have those topological properties.

### 2.4 Population dynamics model

For simplicity, we consider populations with asexual reproduction. (Although a part of the results remain valid for sexual reproduction, as we discuss at the end of this subsection). We choose initial genotypes randomly from a gene pool and assign them to organisms. This choice is invariant with respect to the population member, i.e,. the probability to assign a given genotype *s* to a member of the population does not depend on that member.

In each generation, there are *N*_pop_( *t*) individuals, the genome of each of which is denoted by *s*( *t*), where *t* = 0, 1, 2,... stands for the evolution step number). Following the classical Wright-Fisher ideas, we suppose that generations do not overlap. In each generation (i.e., for each *t*), the following three steps are performed:

1. Each individual *s* at each evolution step can mutate with probability *p*_mut_ per gene;
2. At evolution step *t* each individual with a genotype *s* produces *k* progeny, where *k* is a random non-negative integer, distributed according to the Poisson law
  $$P_{k}=\frac{q^{k}}{k!}\exp(-q),\;(2.8)$$
where *q* = *F*( *s*) is the fitness of that individual;
3. To take into account ecological restrictions on the population size, we introduce the maximal population size *N*_popmax_. If $N^{\prime }$( *t*) > *N*_popmax_, where $N^{\prime }$( *t*) is the number of progeny produced by the population at step *t*, we kill randomly selected individuals in a population-dependent manner. The probability of the death of an individual is given by *p*_kill_( $N^{\prime }$) = 1 *−* ( *N*_popmax_/ $N^{\prime }$( *t*)). If $N^{\prime }$( *t*) *≤ N*_popmax_, we do nothing. We refer to this as the “massacre procedure.”

Conditions 1 and 2 imply that mutations in the genotypes create a new genetic pool and then a new round of selection starts. Condition 3 expresses the fundamental ecological limitation that all environments can only support populations of a limited size. If *N*_popmax_ ≫ 1 then by ( 2.8) and the Central Limit Theorem one can show, under some additional conditions, (see Section 4) that fluctuations of the population size are small, and thus the population is ecologically stable and *N*_pop_( *t*) ≈ *N*_popmax_.

In the limit case of infinitely large populations we will write the discrete dynamical equation for the time evolution of the frequency *X*( *s*, *t*) of the genotype *s* in the population as

$$X(s,t+1)=\bar{F}{\left(t\right)}^{-1}X(s,t)F(s),\;X(s,0)=X_{0}(s),\;(2.9)$$

where $\bar{F}$( *t*) is the average fitness of the population at the moment *t* defined by

$$\bar{F}(t)=\sum_{s\in S(t)}X(s,t)F(s),\;(2.10)$$

where *S*( *t*) is the set of genotypes existing in the population at time *t* (the genetic pool) and *X*( *s*, *t*) = *N*( *s*, *t*) */N*_pop_( *t*) is the frequency of the genotype *s*. Here *N*( *s*, *t*) denotes the number of the population members with the genotype *s* at the step *t*.

The equations ( 2.9) do not take mutations into account. They only describe changes in the genotype frequencies because of selection at the *t*-th time step. The same equations govern evolution in the case of sexual reproduction in the limit of weak selection ^6, 10^. Note that for an evolution defined by ( 2.9), the average fitness $\bar{F}$( *t*) defined by ( 2.10) satisfies Fisher’s theorem, so that this function increases at each time step *t*: $\bar{F}$( *t* + 1) ≥ $\bar{F}$( *t*).

### 2.5 Gene regulation network

In this section, we follow ideas of the classic paper ^29^: the model should include a regulatory network, which evolves itself.

Regulatory genes as well as environmental factors, such as temperature, can influence the trait expression. This effect can be realized via thresholds *h _j_* (we shall describe it below), or via a regulation of coefficients *w _ij_* (see 30). In fact, these approaches are similar for sharp sigmoidal functions *σ* that are close to step functions, as can be shown by ideas from 31. Consider the expression

$$S_{i}=\sum_{i=1}^{N_{g}}w_{ij}s_{j}\;-\;h_{j}\;(2.11)$$

involved in relation ( 2.4). Suppose following 31 that *w _ij_* take the values *γ*, 0 or − *γ*, where *γ* is a parameter, which can be regulated. Let *h _j_* ≪ *γ* be fixed.

Assume that at an evolution step we have *S _i_* > *d _S_*, where *d _S_* > 0 is a parameter, which is more than *γ*. According to our Theorems this fact indicates, that for a well adapted organism the trait *f _j_*( *s*) *≈* 1 and the corresponding coefficient *b _j_* = 1. On the contrary, if *S _i_* < − *d _S_*, then one can expect that *b _j_* = −1 and *f _j_*( *s*) must be 0.

In the both cases, we can regulate the trait expression by a feedback so that whenever *S _i_* attains a critical level, i.e., a trait is well expressed, then *γ* should be increased. In our numerical simulations we use an alternative model, where we change *h _j_*. The alternative model, which is used in our numerical simulations, can be described as follows. We suppose that depending on activity of some regulatory genes or proteins (such as Hsp90), the threshold value can take three values $h_{i}^{\left(-\right)}$, $h_{i}^{\left(+\right)}$ and $h_{i}^{\left(0\right)}$ such that

$$h_{i}^{\left(-\right)}\;\leq \;-D_{h},\;h_{i}^{\left(+\right)}\;\geq \;D_{h},\;\left|h_{i}^{\left(0\right)}\right|\;\approx \;0,\;(2.12)$$

where *D _h_* > 1 is a large parameter that defines the number of genes involved in trait specification (see Subsection 2.2). Thus, *h _j_* can take large negative or positive values, and also a neutral value close to 0. The feedback can be described as follows: if *S _i_* > 0 is large enough and *h _i_* is small, then *h _i_* = − *D _h_*; if *S _i_* < 0 and $\left|S_{i}\right|$ is large enough and *h _i_* is small, then *h _i_* = *D _h_*; otherwise, we do not change *h _i_*.

In our simulations, each Δ *T* evolution steps we modify *h _i_* from 0 to − *D _h_* or *D _h_* for the trait with the maximal value $\left|S_{i}\right|$.

Such a regulation produces a good adaptation even when the number of genes is essentially less than the number of the traits. The plot in Figure 3 shows a difference between an evolution without any regulation and with the regulation by ( 2.12) described above.

**Figure 1.**
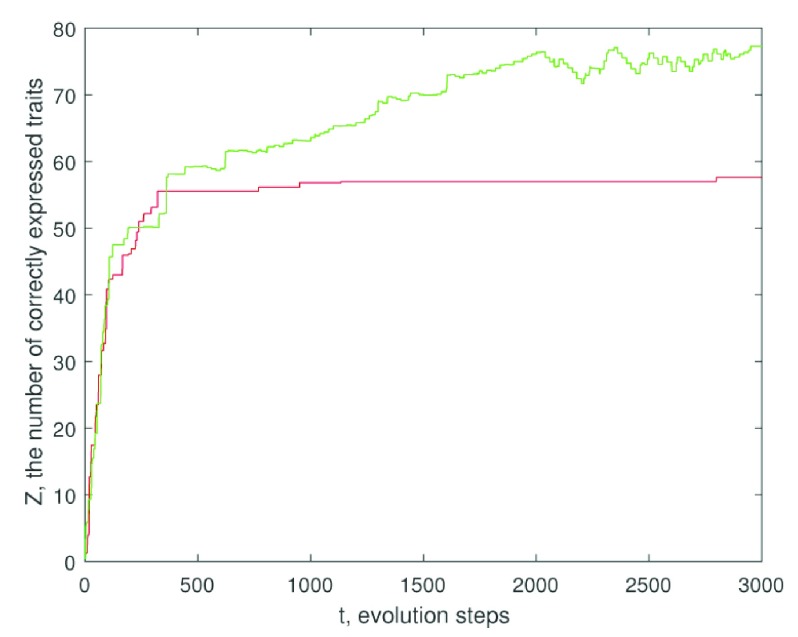
This graph illustrates a difference between the adaptation for evolution without evolution of gene regulation via threshold (the red curve) and with that evolution by 2.12 (the green curve). The parameters are *N _g_* = 50, *M* = 300, the mutation rate *p*_mut_ = 0.01, *γ* = 1 and *K* = 4. Non-zero coefficients *w _ij_* are random numbers distributed according to the standard normal law. The initial genome is a random binary string, where each value is 0 or 1 with probability 1/2. The coefficients *b _i_* are either 1 or −1, where the probability of 1 is *p _b_* = 0.8.

However, the evolution of gene regulation via *h _i_* has an advantage: it makes phenotype more robust. In fact, the traits with large $\left|h_{i}\right|$ are non-sensitive with respect to mutations. The effect produced by this robustness is shown on Figure 1.

**Figure 2.**
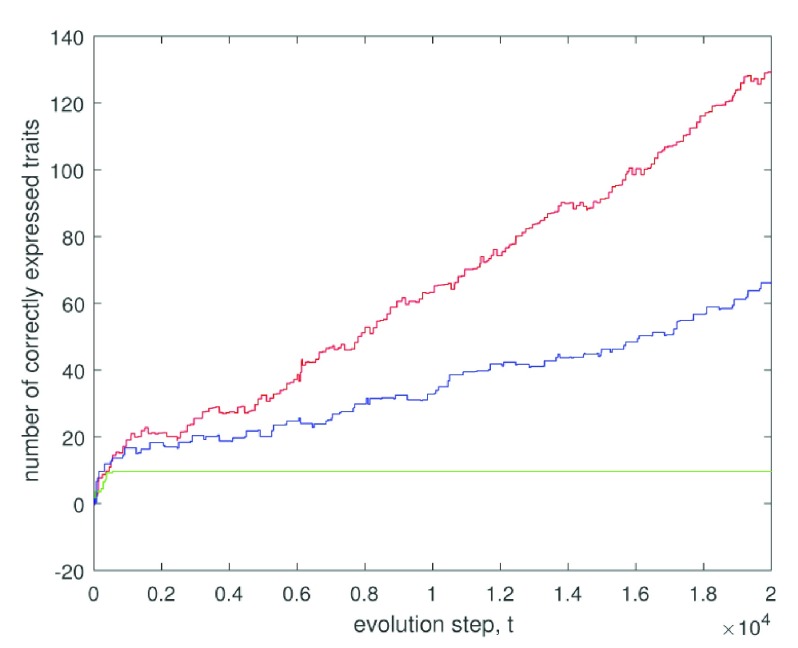
This graph shows that the modular evolution of gene regulation allows adaptation with a few genes, in that case *N _g_* = 10 and the number of traits *M* = 200. This means that we have a very big pleiotropy. Evolution proceeds in 20000 steps. The green curve corresponds to random walk with fixed small *h* without any evolution of gene regulation. We observe that with 10 genes 9 traits are correctly expressed. If evolution goes into 50 rounds then 66 traits are correctly adapted, and if evolution goes in 190 rounds then 129 traits are correctly adapted (the red curve). In that last case, at each step we make adaptation to at most one trait. Parameters are as follows. The mutation rate *p*_mut_ = 0.01 and *K* = 5. Nonzero coefficients *w _ij_* are random numbers distributed according to the standard normal law. The initial genome is a random binary string, where each value is 0 or 1 with probability 1/2. The coefficients *b _i_* are either 1 or –1, where the probability of 1 is *p _b_* = 0.8.

This regulation is even more effective, if we consider the modular evolution, following the recent paper ^32^. Indeed, biological systems are characterized by a high degree of modularity. This modularity allows biological systems to vary only in a small subset of traits at each evolution round. Figure 2 shows an effect of this modularity. We consider a toy example, where a system with only 10 genes should be adapted to 200 constraints. Without regulation, we have no chances to make an adaptation (only about 10 traits are correctly adapted, see the green curve). It is a consequence of a formidable pleiotropy (20 traits on 1 gene). However, if at each evolution stage an organism should be adapted to 4 traits whereas the remaining ones are made robust by high $\left|h_{i}\right|$, then already 66 traits are correctly expressed after 20000 evolution steps. Note that the learning plays a key role in the regulation. In fact, the sign of the regulation threshold *h _i_* depends on the sign of the corresponding coefficient *b _i_*, and the knowledge of that sign gives us important information on the fitness landscape.

**Figure 3.**
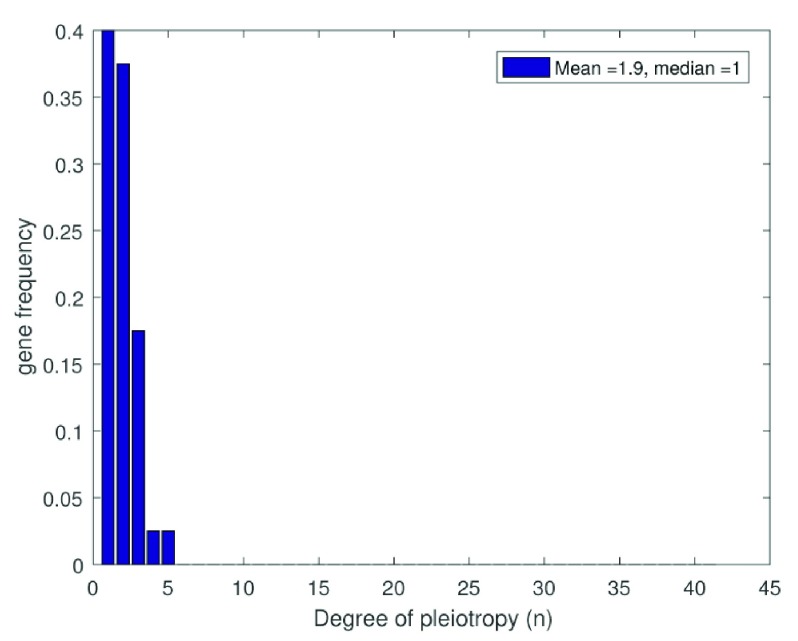
Frequency distributions of degree of gene pleiotropy for model ( 2.4) with the parameters *N _g_* = 4000, *β* = 4, *h* = 0, *N _b_* = 3000.

### 2.6 Adaptation as a hard combinatorial problem

Adaptation (i.e., maximization of fitness in a changing environment) is a very hard problem since over evolutionary history we observe the coevolution of many traits accompanied by changes in many genes. In its general context, this is a problem in the theory of macroevolution, which in general requires the integration of population genetics and developmental biology for its full understanding. There are two key components of this problem. First, development is itself a dynamical process operating over time. Second, there is a combinatorial component of development wherein different combinations of gene must be expressed in different cell types. This combinatorial aspect of the problem means that straightforward theoretical methods of considering the relationship between gene expression and a changing environment that have been very successful in single celled organisms ^33^ cannot be applied to metazoa. In this work, for the sake of tractability, we focus on the combinatorial aspect of the problem and neglect developmental dynamics. Even at the highly simplified level of our model, adaptation is a hard computational problem, as we now demonstrate.

Consider the case, where *f _j_* are defined by relations ( 2.4) and assume that

**i)**  *σ* is the step function;
**ii)**  *b _j_* > 0.

As a consequence of the second assumption, *F* attains its maximum for *f*_1_ = 1, *f*_2_ = 1,..., *f _N _b__* = 1. Let us show that, even in this particular case, the problem of the fitness maximization with respect to *s* is very complex. In fact, for a choice of *h _j_* it reduces to the famous NP-complete problem, so-called *K*-SAT, which has received a great deal of attention in the last few decades (see 34– 39). The *K*-SAT can be formulated as follows.

*K* **-SAT problem.** *Let us consider the set V _n_* = { *s*_1_,..., *s _n_*} *of Boolean variables s _i_* ∈ {0, 1} *and a set _m_ of m clauses. The clauses C _j_ are disjunctions (logical ORs) involving K literals* *z*_*i*_1__, *z*_*i*_2__,..., *z _i _k__, where each z _i_ is either s _i_ or the negation* $\bar{s}$_*i*_ *of s _i_. The problem is to test whether one can satisfy all of the clauses by an assignment of Boolean variables.*

Cook and Levin ^34, 35^ have shown that the *K*-SAT problem is NP-complete and therefore in general it is not feasible in a reasonable running time. In subsequent studies—for instance, by 36 —it was shown that *K*-SAT of a random structure is feasible under the condition that *N _b_ < α _c_*( *K*) *N _g_*, where *α _c_*( *K*) *≈* 2 *^K^* log 2 for large *K*.

The set *_K_* of solutions of random *K*-SAT has a nontrivial structure depending on parameter *α* = *N _b_/N _g_* ^37, 39^. For sufficiently small *α < α _g_*( *K*), where *α _g_*( *K*) *≈* 2 *^K^* log( *K*) */K* is some critical parameter, the set *_K_* forms a giant cluster, where nearest solutions are connected by a single flip and one can go from a solution to another by a sequence of single flips (pointed mutations) ^39^. For *α* ∈ ( *α _g_*, *α _d_*), where *α _d_*( *k*) *< α _c_* is another critical value, solutions form a set of disconnected clusters. The local search algorithms do not work in the domain *α > α _g_*.

Probably, for evolution context *K*-SAT was applied first in 40, where it was used for an investigation of speciation problem.

To see the connection of our model with *K*-SAT, consider equation ( 2.4) supposing that *w _ij_* ∈ {1, 0, −1} and *h _j_* = *−C _j_* + 0.5, where *C _j_* is the number of negative *w _ji_* in the sum $S_{j}=\sum_{i=1}^{N_{g}}w_{ji}s_{i}.$ We set *m* = *N _b_* and *n* = *N _g_*. Under this choice of *h _j_*, the terms *σ*( *S _j_*) can be represented as disjunctions of literals *z _j_*. Each literal *z _j_* equals either *s _j_* or $\bar{s}$ *_j_*, where $\bar{s}$ *_j_* denotes negation of *s _j_*. To maximize the fitness, we must assign *s _j_* such that all disjunctions will be satisfied. If we fix the number *n _j_* of the literals participating in each disjunction (clause) and set *n _j_* = *K*, this assignment problem is precisely the *K*-SAT problem formulated above.

Reduction to the *K*-SAT problem is a transparent way of representing the idea that multiple constraints need to be satisfied. The number *K* defines the gene redundancy and the probability of gene pleiotropy. Remind that pleiotropy occurs when one gene influences two or more seemingly unrelated phenotypic traits. The threshold *h _j_* and *K* define the number of genes which need be flipped in order to attain a high expression of the trait *f _j_*. Note that gene pleiotropy is a fundamental characteristics ^41^, which is studied for real organisms only recently (see 19). We can compare experimental observations and consequence of model ( 2.4), which is a generalisation of *K*-SAT (compare plot Figure 3 and plots on Figure 1 in 19). So, we can fit our model parameters using real data. Moreover, we can check validity of our model by the following arguments.

We note that, in the case of giant cluster formation, the topological properties of the solution set *_K_*, mentioned above, outline the properties of really observed fitness landscapes ^22^: existence of many peaks, valleys and ridges connecting peaks. Namely, existence of many solutions of *K*-SAT, when a giant cluster exists, means that the landscape has a number of peaks separated by valleys. On the other hand, connectance of solutions within the giant cluster can be interpreted that there exist ridges that connect peaks.

Note that there are important differences between *K*-SAT in Theoretical Computer Science and fitness maximization problems. First, the signs of *b _j_* are unknown for real biological situations since the fitness landscape is unknown. Second, our adaptation problem involves the threshold parameters *h _j_* (see ( 2.4)). In contrast to *K*-SAT, in our case the Boolean circuit is plastic, because the *h _j_* are not fixed.

If the *b _j_* are unknown, the adaptation (fitness maximization) problem becomes even harder because we do not know the function to optimize. Therefore, many algorithms for *K*-SAT are useless for biological adaptation problems. Below we will nonetheless obtain some analytical results based on the assumption that *b _j_* are random.

## 3. Main theorems

The subsequent material is organized as follows. First we formulate a result on regulation mechanism power. Furthermore, we prove two fitness landscape learning theorems.

### 3.1 Fitness landscape learning theorems

For simplicity, we consider asexual reproduction. To obtain similar results for sexual reproduction, one can consider a weak selection regime and use the results of 10, where eq. ( 2.9) are derived.

Let us introduce two sets of indices *I*_+_ and *I _−_*, such that *I*_+_ ∪ *I _−_* = {1,..., *N _b_*}. We refer to these sets in the sequel as positive and negative sets, respectively. We have

$$I_{+}=\left\{j\in \left\{1,\ldots ,N_{b}\right\}|b_{j}>0\right\},\;(3.1)$$

$$I_{-}=\left\{j\in \left\{1,\ldots ,N_{b}\right\}|b_{j}<0\right\}.\;(3.2)$$

The biological interpretation of that definition is transparent: the expression of the traits *f _j_* with *j* ∊ *I*_+_ increases the fitness and for *j* ∊ *I*_–_ expression of the trait decreases the fitness.

Let *s* and $\bar{s}$ be two genotypes. Then we denote by Diff( *s*, $\bar{s}$) the set of positions *i* such that *s _i_* ≠ $\bar{s}$ *_i_*:

$$\mathrm{Diff}\text{(}s,\bar{s}\text{)}=\left\{i\in \left\{\text{1,}\ldots ,N_{g}\right\}|s_{i}\neq \bar{s}\right\}\cdot$$

The set Diff( *s*, $\bar{s}$) indicates which genes in *s* should be flipped in order to obtain $\bar{s}$.

We formulate two theorems on fitness landscape learning. First we consider the case of infinitely large populations.

**Theorem 3.1.** *Suppose that the evolution of the genotype frequencies X*( *s*, *t*) *is determined by equations ( 2.9) and ( 2.10). Moreover, assume that*

**I** *for all t* ∊ [ *T*_1_, *T*_1_ + *T _c_*] *, where T*_1_, *T _c_ >* 0 *are integers, the population contains two genotypes s and* $\bar{s}$ *such that the frequencies X*( *s*, *t*) *and X*( $\bar{s}$, *t*) *satisfy*

$$X(s,T_{1})=p_{0}>0,\;X\text{(}\bar{s},T_{1}+T_{C}\text{)}=p_{\text{1}}>0,\;(3.3)$$

**II** *we have*

$$\text{Diff(}s\text{,}\bar{s}\text{)}\subset K_{j,}\;(3.4)$$

*for some j. In other words, the genes s _i_ such that s _i_* ≠ $\bar{s}$ *_i_* *are involved in a single regulation set K _j_; and finally,*

**III** *Let*

$$\delta_{j}=|f_{j}(s)-f_{j}(\bar{s})|>0,\;|b_{j}|>0,\;(3.5)$$

*and*

$$T_{c}>\frac{-\log(p_{0}p_{1})}{|b_{j}|\delta_{j}}\cdot \;(3.6)$$

*Then, if*

$$f_{j}(s)<f_{j}(\bar{s}),\;(3.7)$$

*we have j* ∊ *I*_+_. *If f _j_*( *s*) > *f _j_*( $\bar{s}$), *then j* ∊ *I _–_*.

Before proving this, let us make some comments. The biological meaning of the theorem is simple: for simple fitness models, where unknown parameters *b _j_* are involved in a linear way, in the limit of infinitely large populations fitness landscape learning is possible.

Moreover, note that we do not make any specific assumptions about the nature of mutation, but only that all genetic variation between *s* and $\bar{s}$ are contained in a single regulatory set *K _j_*.

The assertion of Theorem 3.1 is not valid if the set Diff( *s*, $\bar{s}$) belongs to a union of different regulation sets *K _j_ , j* = *j*_1_, . . . , *j _p_* with *p* > 1. This effect of belonging to different sets *K _j_* is pleiotropy in gene regulation. Note that if *N _b_* ≪ *N _g_* then the pleiotropy probability is small for large genome lengths *N _g_*. On the contrary, if *N _b_* ≫ *N _g_* then assumption **II** is invalid.

Assumption **II** looks natural if when we deal with point mutations. In fact, if $\bar{s}$ is obtained from *s* by a single point mutation then condition ( 3.4) always holds for some *j*. For small mutation rates the probability of two point mutations is essentially below than the probability of a single mutation.

To conclude let us note that Theorem gives a rough estimate for the learning time *T _c_*:

$$T_{c}=O(-\frac{\log(p_{0}p_{1})}{|b_{j}|\delta_{j}})\cdot$$

*Proof.* The main idea is simple. Negative mutations lead to elimination of mutant genotypes from the population, and the corresponding frequencies become, for large times, exponentially small.

Assume that ( 3.7) holds. Let *j ∊ I _−_*, and thus *b _j_ <* 0. Consider the quantity

$$Q(t)=\frac{X(s,t)}{X(\bar{s},t)}=\frac{N(s,t)}{N(\bar{s},t)}\cdot \;(3.8)$$

According to assumption **II**

$$\Delta W=W(s)-W(\bar{s})=b_{j}(f_{j}(s)-f\text{(}\bar{s}\text{)})\cdot \;(3.9)$$

Assumption **III** entails that

$$\Delta W\geq |b_{j}|\delta_{j\cdot }\;(3.10)$$

Relations ( 2.6) and ( 3.10) imply

$$\frac{F(s)}{F(\bar{s})}=\exp(\Delta W)\geq \exp(|b_{j}|\delta_{j})\cdot$$

By ( 2.9) and the last inequality we find that for *T > T*_1_

$$Q(T)\geq Q(T_{1})\exp(|b_{j}|\delta_{j}(T-T_{1}))\cdot \;(3.11)$$

Consider inequality ( 3.11) for *T* = *T*_1_ + *T _c_*. Let us note that in the relation *Q*( *T*_1_) = *X*( *s*, *T*_1_) */X*( $\bar{s}$, *T*_1_) the numerator is *p*_0_ whereas the denominator ≤ 1. Thus, *Q*( *T*_1_) ≥ *p*_0_. The same arguments show that *Q*( *T*_1_ + *T _c_* ) ≤ 1 */p*_1_. Therefore, by ( 3.11) one obtains that

$$\frac{1}{p_{1}p_{0}}\geq \exp(|b_{j}|\delta_{j}T_{c})\cdot \;(3.12)$$

This inequality leads to a contradiction for *T _c_* satisfying ( 3.6), thus completing the proof.

### 3.2 The case of finite populations

Theorem 3.1 can be extended to the case of finite populations and non-zero mutation rates. is small. To formulate this generalization, we need an additional assumption about the fitness function. Suppose that

$$\;\begin{array}{l} 1<c_{F}\;<\;\min\;F(s),\\ \;s\in S(t) \end{array}$$

$$\begin{array}{l} \max F(s)\;<\;C_{F},\;\forall t\in \text{[}T_{\text{1}}\text{,}\;T_{\text{1}}+T_{c}\text{]}\\ s\in S(t) \end{array}\;(3.13)$$

where *c _F_*, *C _F_ >* 0 are constants independent of *t*. For example, if

$$\sum_{j=1}^{N_{b}}\left|b_{j}\right|<\gamma ,$$

then *c _F_* = *K _F_* exp(– *γ*) and *C _F_* = *K _F_* exp( *γ*) and ( 3.13) holds if *K _F_* > exp( *γ*).

Condition ( 3.13) means that each individual gives birth to at least *c _F_* and at most *C _F_* descendants, where those bounds do not depend on the population size and evolution step.

Let

$$N_{\text{pop}}(T_{1})=N_{\text{popmax}}\cdot \;(3.14)$$

Note that for simplicity in the next Theorem 3.2 we consider point mutations (bit flipping) only. The model used here cannot represent mutations of arbitrarily small effect, but it can include insertions or deletions. In contrast to Theorem 3.2, Theorem 3.1 is valid for all kinds of mutations.

Then we have

**Theorem 3.2.** *Consider the population dynamics defined by model* **1** *-* **3** *in Subsection 2.4. Assume conditions* ( 3.14) *and* **M** *hold, and assumptions ( 3.3), ( 3.4), ( 3.5), ( 3.7) of Theorem 3.1 are satisfied. Suppose*

$$X(s,t)\geq p_{0}\;\forall t\in \text{[}T_{\text{1}}\text{,}\;T_{\text{1}}\;\text{+}\;T_{c}\text{],}\;(3.15)$$

$$X(\bar{s},t)\geq p_{1}\;\forall t\in \text{[}T_{\text{1}}\text{,}\;T_{\text{1}}\;\text{+}\;T_{c}\text{]}\cdot \;(3.16)$$

*Then if j* ∊ *I _–_ the inequality*

$$\;p_{1}<p_{0}^{-1}\exp(-0.5|b_{j}|\delta_{j}T_{c})\;(3.17)$$

*is fulfilled with the probability* Pr *_v_ such that*

$${\mathrm{Pr}}_{v}>{\left(1-\rho (p_{\text{0}})-\rho (p_{\text{1}})\right)}^{T_{c}},\;(3.18)$$

*where for large N _popmax_ and p _mut_* → 0

$$\rho (p)=\exp\left(-(\ln2-1/2)p_{\text{mut}}c_{F}p\kappa N_{\mathrm{popmax}}\right)\cdot$$

*Interpretation of Theorem 3.2*

It is interesting to compare Theorem 3.1 and Theorem 3.2. The previous one asserts that for infinite populations the probability of the event *j ∊ I _−_* is zero whereas the second one claims that this probability becomes exponentially small as the population size increases.

This theorem also shows that evolution can make a statistical test checking the hypothesis *H _–_* that *j ∊ I*_–_ against the hypothesis *H*_+_ that *j ∊ I*_+_. Suppose that *H*_–_ is true. Let *V* be the event that the frequency *X*( $\bar{s}$, *t*) of the genotype $\bar{s}$ in the population is larger than *p*_1_ within a sufficiently large time *T _c_*. According to estimate ( 3.18), the probability of the event *V* is so small that it is almost unbelievable. Therefore, the hypothesis *H*_–_ should be rejected. We will refer *T _c_* as the *checking time*.

*Rare mutants*. In this Theorem we assume that the frequencies *p*_0_ and *p*_1_ of genotypes (wild and mutant) are fixed and our estimate is valid as *p*_mut_ → 0. I.e., we do not consider mutants with a very small frequencies (fractions). Of course, a large population always contains a small number of such mutants. In numerical simulations we assume that evolution is successful and population is perfectly adapted, if, say, 95 or 99 percents of population members have the maximal fitness.

*Ideas for the proof*. The main idea is the same as that for the previous theorem: we compare the frequencies of the organisms with the genotype $\bar{s}$ and the organisms with the genotype *s*. However, the proof includes a number of technical details connected with estimates of mutation effects and fluctuations. The formal proof can be found in Section 4. It is based on estimates of the accuracy of the Nagylaki equations ( 2.9). The main Lemma 4.5 for the proof of Theorem 3.2. admits a transparent interpretation. We show that fraction *X*( *s*, *t*) of genotype *s* evolves in time in such a way that the estimates.

$$X(s,t+1)\;\text{​}\;<\;(F(s)+r(p_{\text{mut}},\text{​}\;N_{\text{popmax}},\;s))X(s,\;t)/\;\bar{F}(t)$$

$$X(s,t+1)\;\text{​}\;>\;(F(s)-r(p_{\text{mut}},\text{​}\;N_{\text{popmax}},\;s))X(s,\;t)/\;\bar{F}(t)$$

are satisfied, where *F*( *s*) is a fitness of genotype *s*, $\bar{F}$ is average population fitness, and *r*( *p _mut_*, *N _pop_*)) are small corrections, which converge to zero uniformly in *s* as the mutation rate *p*_mut_ → 0 and the population size *N*_popmax_ → ∞. This means that in the limit *p*_mut_ → 0, *N*_popmax_ → ∞ we have equation ( 2.9). The main problem with the application of Theorem 3.2 is how it allows to perform fitness landscape learning. It can be done by a regulation, as is detailed in the following section.

## 4 Proof of theorems

Let us prove Theorem 3.2.

### 4.1 Main tools and auxiliary Lemmas

Let us introduce notation and make some preliminary remarks. Remind that we denote by *N*( *s*, *t*) the number of the population members with the genotype *s* at the moment *t*. Let *X*( *t*) be the set of all population members at the moment *t*. For each *x ∊ X*( *t*) let us denote by $N^{\prime }$ ( *x*, *t*) the number of progeny born by the individual *x* at the moment *t* before the massacre (see point 3 of model from Subsection 2.4). Let *s _g_* ( *x*) be the genotype of *x*. Then, according to ( 2.8), the mean of $N^{\prime }$( *x*, *t*) is

$$EN^{\prime }\left(x,t\right)=F\left(s_{g}\left(x\right)\right),\;(4.1)$$

where *EX* denotes the expected value of *X*. By $\overset{-}{N}$ ( *s*, *t*) we denote the number of all progeny born by individuals with the genotype *s* at the moment *t* before the massacre. Since all progeny are produced independently and randomly, the previous relation gives

$$E\bar{N}\left(s,t\right)=N\left(s,t\right)F\left(s\right).\;(4.2)$$

Our main analytical tools are the Chernoff bounds and the Hoeffding inequalities. We also use the Markov inequality: for a positive random quantity *X* and *a >* 0 one has

$$Pr\left\{X>a\right\}\leq \frac{EX}{a}.\;(4.3)$$

Moreover, we use two elementary estimates. Let be an event in stochastic population dynamics. We denote by *Not* the negation (complement) of ** and by Pr( *|ℬ*) the conditional probability of ** under the condition *ℬ*. For events ** , *ℬ*_1_, . . . , *ℬ _n_* we have

$$\begin{array}{l} \text{Pr}\left(A\right)=\text{Pr}\left(A{ℬ}_{1}\ldots \;{ℬ}_{n}\right)+\\ \;\text{Pr}\left(A\text{Not}\;\left({ℬ}_{\text{1}}\ldots \;{ℬ}_{n}\right)\right)\\ \;\leq \text{Pr}\left(A|{ℬ}_{1}\ldots \;{ℬ}_{n}\right)+\sum_{j=1}^{n}\text{Pr}(\text{Not}\;{ℬ}_{j}).\;(4.4) \end{array}$$

For two events **, *ℬ* one has

Pr(Aℬ)≥1−Pr(NotA)−Pr(Notℬ).(4.5)

**Lemma 4.1.** *Let X _i_ be independent random quantities, where i* = 1, . . . , *n. Let each X _i_ be distributed according to the Poisson law with the average *EX _i_* = μ _i_. Let us denote*

$$X=\sum_{j=1}^{n}X_{j},\;\bar{\mu }=\frac{\sum_{j=1}^{n}\mu_{j}}{n}.$$

*Then for all δ >* 0

$$\text{Pr}\left\{X>\left(1+\delta \right)\bar{\mu }n\right\}\leq \text{exp}\left(-\bar{\mu }d\left(\delta \right)n\right),\;(4.6)$$

*where*

d(δ)=(1+δ)ln(1+δ)−δ.

*Similarly,*

$$\text{Pr}\left\{X<\left(1-\delta \right)\bar{\mu }n\right\}\leq \text{exp}\left(-\bar{\mu }d\left(-\delta \right)n\right).\;(4.7)$$

**Proof**. Note that for any *λ >* 0

$$\begin{array}{l} \text{Pr}\left\{X>\left(1+\delta \right)\bar{\mu }n\right\}=\\ \;\text{Pr}\left\{\text{exp}\left(\lambda X\right)>\text{exp}\left(\lambda \left(1+\delta \right)\bar{\mu }n\right))\}\;(4.8\right) \end{array}$$

Since *X _j_* are independent quantities, we have

$$E\;\text{exp}\left(\lambda X\right)=\prod_{j=1}^{n}E\;\text{exp}\left(\lambda X_{j}\right).$$

The straight forward computation shows that

$$E\text{exp}\left(\lambda X_{j}\right)=\text{exp}\left((e^{\lambda }-1)\mu_{j}\right).$$

Therefore, due to the Markov inequality ( 4.3) and estimate ( 4.8) one has

$$\text{Pr}\left\{X>\left(1+\delta \right)\bar{\mu }n\right\}\leq \text{exp}\left(n\bar{\mu }f(\lambda )\right),$$

where

f(λ)=exp(λ)−1−λ(1+δ).

We minimize *f* with respect to *λ* and obtain ( 4.6). To derive ( 4.7), we use

$$\begin{array}{l} \text{Pr}\left\{X<\left(1-\delta \right)\bar{\mu }n\right\}=\\ \;\text{Pr}\{\text{exp}\left(-\lambda X\right)>\\ \;\text{exp}\left(-\lambda \left(1-\delta \right)\bar{\mu }n\right))\}\;(4.9) \end{array}$$

and repeat the same arguments. The Lemma is proved.

**Lemma 4.2.** *Let X _i_ be independent random quantities, where i* = 1, . . . , *n such that X _i_ ∊* {0, 1} *and EX _i_* = *p. Then*

Pr{2X<pn}≤exp(−g(p)n),(4.10)

*where*

$$g\left(p\right)=-\frac{p\;\text{ln}2}{2}-\text{ln}(1-\frac{p}{2}).\;(4.11)$$

**Proof**. Note that for any *λ >* 0

Pr{X<pn/2}=Pr{exp(−λX)>exp(−λpn/2))}.(4.12)

Since *X _j_* are independent quantities, we have

$$E\;\text{exp}\left(-\lambda X\right)=\prod_{j=1}^{n}E\;\text{exp}\left(-\lambda X_{j}\right).$$

Note that *E* exp( *−λX _j_*) = *p* exp( *−λ*) + 1 *− p*. Let

G(λ,p)=λp/2+ln(pexp(−λ)+1−p).

We take *λ* = ln 2 and find that *G*(ln 2, *p*) = *−g*( *p*). Now by using the Markov inequality ( 4.3) and estimate ( 4.12) one obtains ( 4.10). The Lemma is proved.

We also use the following Chernoff-Hoeffding theorem. Let *X _i_* be i.i.d. quantities such that *X _i_* ∊ {0, 1} and *EX _i_* = *p*, where *i* = 1, . . . , *n*. Then for $X=\sum_{j=1}^{n}X_{j}$ one has

Pr{X>(p+ε)n}≤exp(−D(p+ε‖p)n),(4.13)

where *D*( *x||y*) is the Kullback-Leibler divergence

D(x‖y)=xln(x/y)+(1−x)ln((1−x)/(1−y)).(4.14)

Moreover, we will use the Hoeffding Theorem: if i.i.d. quantities *X _i_ ∊* [0, 1] with the probability 1 then

$$\text{Pr}\left\{\left|X-EX\right|>a\right\}\leq 2\;\text{exp}\left(-2a^{2}/n\right).\;(4.15)$$

### 4.2 Main lemmas

First we estimate the population size fluctuations.

**Lemma 4.3**. *Let* $\bar{N}$( *t*) *be the number of all progeny, born in the population at the moment t before the massacre, and ε*_1_ > 0 *be a small number. Then*

$$\begin{array}{l} \;\bar{N}\left(t\right)\in J_{\varepsilon_{1}}\left(t\right)=\\ \left[\left(1-\varepsilon_{1}\right)\bar{F}\left(t\right)N_{\text{pop}}\left(t\right),\;\left(1+\varepsilon_{1}\right)\bar{F}\left(t\right)N_{\text{pop}}\left(t\right)]\;(4.16\right) \end{array}$$

*with probability*

$${\text{Pr}}_{\bar{N}}>1-\eta_{0}\left(\varepsilon_{1}\right),\;(4.17)$$

*where*

$$\begin{array}{l} \eta_{0}\left(\varepsilon_{1}\right)=\text{exp}\left(-d\left(\varepsilon_{1}\right)c_{F}N_{\text{pop}}\left(t\right)\right)+\\ \;\text{exp}\left(-d\left(-\varepsilon_{1}\right)c_{F}N_{\text{pop}}\left(t\right)\right).\;(4.18) \end{array}$$

*Proof.* Let $n^{\prime }$ ( *x*, *t*) denote the number of progeny produced by the individual *x* before the massacre at the *t*-th evolution step. The number $\bar{N}$( *t*) is the sum

$$\bar{N}\left(t\right)=\sum_{x\in X\left(t\right)}N^{\prime }\left(x,t\right)$$

of the mutually independent random quantities. According to ( 4.2), the average *E* $N^{\prime }$ ( *x*, *t*) is *F*( *s _g_* ( *x*)). Therefore,

$$E\bar{N}\left(t\right)=\sum_{x\in X\left(t\right)}EN^{\prime }\left(x,t\right)\;(4.19)$$

$$\;=\sum_{x\in X\left(t\right)}F\left(s_{g}\left(x\right)\right)\;(4.20)$$

$$\;=N_{\text{pop}}\left(t\right)\bar{F}\left(t\right).\;(4.21)$$

We set

$$n=N_{\text{pop}}\left(t\right),\;\mu_{x}=F\left(s_{g}\left(x\right)\right),\;\bar{\mu }=\bar{F}\left(t\right)$$

and use the Lemma 4.1 that gives us ( 4.17).

**Lemma 4.4.** *Let* *ε*_2_ ∊ (0, 1) *be fixed and condition* ( 3.13) *be fulfilled. Assume, moreover, that*

$$2N_{\text{popmax}}\geq N_{\text{pop}}\left(t\right)\geq \kappa N_{\text{popmax}},\;(4.22)$$

*where*

$$\kappa \in \left(c_{F}^{-1},\;1\right)\;(4.23)$$

*and c _F_* > 1 *is defined by ( 3.13). Let us define the event *_*ε*_2__ ( *t*) *by*

$${}_{\varepsilon_{2}}\left(t\right)=\left\{\left|N_{\text{pop}}\left(t+1\right)-N_{\text{popmax}}\right|<\varepsilon_{2}N_{\text{popmax}}\right\}.\;(4.24)$$

*Then one has*

$$\text{Pr}\left({}_{\varepsilon_{2}}\left(t\right)\right)>1-\eta \left(\varepsilon_{2}\right),\;(4.25)$$

*where*

$$\begin{array}{l} \;\eta \left(\varepsilon_{2}\right)=\\ \;\text{exp}\left(-\;d\left(\tilde{\varepsilon }\right)\kappa N_{\text{popmax}}\right)+\\ \text{exp}\left(-\;d\left(-\tilde{\varepsilon }\right)\kappa N_{\text{popmax}}\right)+\\ \;2\;\text{exp}\left(-\frac{2\varepsilon_{2}^{2}N_{\text{popmax}}}{2\left(1+\tilde{\varepsilon }\right))C_{F}})\;(4.26\right) \end{array}$$

*and*

$$\tilde{\varepsilon }=1-{\left(\kappa c_{F}\right)}^{-1}.\;(4.27)$$

*Proof.* Let *ξ*( *x*) be random quantities defined as follows: *ξ*( *x*) = 1 if the individual *x* is survived as a result of massacre (see point 3 of our model from Subsection 2.4), and *ξ*( *x*) = 0 otherwise. Let $X^{\prime }$ ( *t*) be the set of progeny produced by all individuals from the population. Then the number *N*_sur_( *t*) = *N*_pop_( *t* + 1) of finally survived progeny can be computed as follows:

$$N_{\text{sur}}\left(t\right)=\sum_{x\in X^{\prime }\left(t\right)}\xi \left(x\right).$$

Note that *| $X^{\prime }$*( *t*) *|* = $\bar{N}$( *t*). Moreover, *Eξ*( *x* ) = *N*_popmax_/ $\bar{N}$( *t*) for $\bar{N}$( *t*) *≥ N*_popmax_. Therefore, if $\bar{N}$( *t*) *≥ N*_popmax_ then

$$EN_{\text{sur}}\left(t\right)=N_{\text{popmax}}.\;(4.28)$$

Let us define the event

$$ℬ\left(t\right)=\left\{\bar{N}\left(t\right)\in J_{\tilde{\varepsilon }}\left(t\right)\right\},\;(4.29)$$

where the interval *J _ε_*( *t*) is defined by ( 4.16) and $\tilde{\varepsilon }$ is defined by ( 4.27). By ( 4.4) we have

$$\begin{array}{l} \text{Pr}\left(\text{Not}\;{}_{\varepsilon_{2}}\left(t\right)\right)\leq \text{Pr}\left(Not\;{}_{\varepsilon_{2}}\left(t\right)|ℬ\left(t\right)\right)+\\ \;\text{Pr}\left(\text{Not}\;ℬ\left(t\right)\right).\;(4.30) \end{array}$$

Now we apply the Hoeffding inequality ( 4.15). For each *ε*_2_ > 0 we obtain

$$\text{Pr}\left(\text{Not}\;{}_{\varepsilon_{2}}\left(t\right)\right)<2\;\text{exp}(-\frac{2\varepsilon_{2}^{2}EN_{\text{sur}}{\left(t\right)}^{2}}{\bar{N}\left(t\right)}).$$

If *ℬ* ( *t*) takes place, then $\bar{N}$( *t*) *≥ N*_popmax_ and consequently

$$\frac{2\varepsilon_{2}^{2}EN_{\text{sur}}{\left(t\right)}^{2}}{\bar{N}\left(t\right)}>\frac{2\varepsilon_{2}^{2}N_{\text{popmax}}}{2\left(1+\tilde{\varepsilon }\right)C_{F}}.\;(4.31)$$

Therefore,

$$\text{Pr}\left(Not\;{}_{\varepsilon_{2}}\left(t\right)|ℬ\left(t\right)\right)<2\;\text{exp}(-\frac{2\varepsilon_{2}^{2}N_{\text{popmax}}}{2\left(1+\tilde{\varepsilon }\right)C_{F}}).\;(4.32)$$

Moreover, by Lemma 4.3

$$\begin{array}{l} \text{Pr}\left(\text{Not}\;ℬ\left(t\right)\right)<\text{exp}\left(-d\left(\tilde{\varepsilon }\right)\kappa N_{\text{popmax}}\right)+\\ \;\text{exp}\left(-d\left(-\tilde{\varepsilon }\right)\kappa N_{\text{popmax}}\right).\;(4.33) \end{array}$$

Inequalities ( 4.30), ( 4.32) and ( 4.33) prove ( 4.25).

The following lemma, in particular, allows us to obtain equations ( 2.9) and ( 2.10) in the limit of infinite populations and for small mutation probabilities.

Recall that $\bar{N}(s,\;t)$ denotes the number of non-mutated progeny generated by the individuals with the genotype *s* before the massacre. Let *N*_sur_( *s*, *t*) be the number of those progeny that survived after that massacre.

**Lemma 4.5.** *Let ε*_0_ *be a positive number satisfying* ( 4.75) *and*

$$\kappa N_{\text{popmax}}<N_{\text{pop}}(t)<2N_{\text{popmax}}.\;(4.34)$$

*Then one has*

$$N(s,t+1)>(1-\varepsilon_{0})F(s)\bar{F}{\left(t\right)}^{-1}N(s,t)\;(4.35)$$

*with the probability* Pr _*s*, *t*,+_ *such that*

$${\text{Pr}}_{s,t,+}>1-\sum_{i=1}^{5}R_{i}(s,t),\;(4.36)$$

*where*

$$\begin{array}{l} R_{1}(s,\;t)=\text{exp}(-d(1)c_{F}N_{\text{pop}}(t))+\\ \;\text{exp}(-d(-1)c_{F}N_{\text{pop}}(t)), \end{array}\;(4.37)$$

$$\begin{array}{l} R_{2}(s,\;t)=\text{exp}(-d(0.5)c_{F}N(s,t))+\\ \;\text{exp}(-d(-0.5)c_{F}N(s,t)), \end{array}\;(4.38)$$

$$R_{3}(s,\;t)=2\text{exp}\left(\frac{-\varepsilon_{0}^{2}}{16C_{F}^{2}}c_{F}N(s,t)\right),\;(4.39)$$

$$\begin{array}{l} R_{4}(s,\;t)=\text{exp}(-0.5(2\;\text{ln}\;2\;p_{\text{mut}}+\\ \;(1-p_{\text{mut}})\;\text{ln}(\frac{1-2p_{\text{mut}}}{1-p_{\text{mut}}}))\cdot \\ \;c_{F}N(s,t)),\;(4.40) \end{array}$$

$$\begin{array}{l} R_{5}(s,\;t)=\text{exp}(-d(1)c_{F}N(s,t))+\\ \;\text{exp}(-d(-1)c_{F}N(s,t)),\;(4.41) \end{array}$$

*Similarly,*

$$N(s,t+1)<(1+\varepsilon_{0})F(s)\bar{F}{\left(t\right)}^{-1}N(s,t)\;(4.42)$$

*with the probability* Pr _*s*, *t*,−_ *such that*

$${\text{Pr}}_{s,t,-}>1-\sum_{i=1}^{5}R_{i}(s,t).\;(4.43)$$

*Proof. Step 1, estimates of fluctuations*. First let us estimate the fluctuations of the number $\bar{N}(s,\;t)$. For each *ε*_2_ > 0 let us define the event

$$A_{s,\varepsilon_{2}}(t)=\{|\bar{N}(s,t)-E\bar{N}(s,t)|>\varepsilon_{2}E\bar{N}(s,t)\}.\;(4.44)$$

By Lemma 4.1 one has

$$\begin{array}{l} \text{Pr}(A_{s,\varepsilon_{2}}(t))<\text{exp}(-d(\varepsilon_{2})E\bar{N}(s,t))+\\ \;\text{exp}(-d(-\varepsilon_{2})E\bar{N}(s,t)). \end{array}\;(4.45)$$

Note that

$$E\bar{N}(s,t)=F(s)N(s,t)>c_{F}N(s,t).\;(4.46)$$

As a result, by ( 4.46) we obtain

$$\begin{array}{l} \text{Pr}(A_{s,\varepsilon_{2}}(t))<\text{exp}(-d(\varepsilon_{2})c_{F}N(s,t))+\\ \;\text{exp}(-d(-\varepsilon_{2})c_{F}N(s,t)).\;(4.47) \end{array}$$

*Step 2.* Here we estimate the number of progeny that survived as a result of the massacre procedure (point 3 of the population dynamics model, see subsection 2.9). Let *X'* ( *s, t*) be the set of progeny produced by individuals with the genotype *s*. Then the number *N*_sur_( *s, t*) of survived progeny *x* for individuals *x* belonging to the set *Z'* ( *s, t*) is

$$N_{\text{sur}}(s,t)=\sum_{x\in X^{\prime }(s,t)}\xi (x),$$

where *ξ*( *x*) are defined in the proof of the previous Lemma. For *ε*_3_ > 0 we consider the event

$$\begin{array}{l} A_{\text{sur,}s,\varepsilon_{\text{3}}}(t)=\{|N_{\text{sur}}(s,t)-EN_{\text{sur}}(s,t)|\\ \;\text{>}\;\varepsilon_{\text{3}}E[N_{\text{sur}}(s,t)]\},\;\;(4.48) \end{array}$$

Let us estimate the probability Pr( _sur, *s*_( *t*)). According to the Hoeffding Theorem ( 4.15)

$$\begin{array}{l} \;\text{Pr}\left(A_{\text{sur},s,\varepsilon_{3}}\left(t\right)\right)<\\ 2\;\text{exp}\left(-2\varepsilon_{3}^{2}E{\left[N_{\text{sur}}\left(s,t\right)\right]}^{2}\bar{N}{\left(s,t\right)}^{-1}\right).\;(4.49) \end{array}$$

Note that *ξ*( *x*) and *ξ*( *y*) are independent quantities for different *x* and *y*, thus under the condition $\bar{N}(t)$ > *N*_popmax_

$$EN_{\text{sur}}(s,t)=\sum_{x\in X^{\prime }(s,t)}E\xi (x)=\bar{N}(s,t)\frac{N_{\text{popmax}}}{\bar{N}(t)},$$

therefore,

$$\text{Pr}(A_{\text{sur},s,\varepsilon_{3}}(t))<2\;\text{exp}(-2\varepsilon_{3}^{2}\bar{N}(s,t)(\frac{N_{\text{popmax}}}{\bar{N}(t)}{)}^{2}).\;(4.50)$$

Let us define the events *ℬ*_*s*_( *t*) and *ℬ*( *t*) by

$$\begin{array}{l} {ℬ}_{s}(t)=\{\bar{N}(s,t)\\ \;>(1-\varepsilon_{2})E\bar{N}(s,t)\},\;(4.51) \end{array}$$

$$\begin{array}{l} ℬ(t)=\{\bar{N}(t)\\ \;<(1+\varepsilon_{1})E\bar{N}(t)\}.\;(4.52) \end{array}$$

Then using ( 4.4) one has

$$\;\begin{array}{l} \mathrm{Pr}(A_{\text{sur},s,\varepsilon_{3}}(t))\;\leq \;\mathrm{Pr}(A_{\text{sur},s}(t)|{ℬ}_{s}(t)ℬ(t))+\\ \;\mathrm{Pr}(\text{Not}\;{ℬ}_{s}(t))+\\ \;\mathrm{Pr}(\text{Not}\;ℬ(t)).\;(4.53) \end{array}$$

We observe that under conditions ℬ _*s*_( *t*) and ℬ( *t*)

$$\begin{array}{l} \bar{N}(s,t){\left(\frac{N_{\text{popmax}}}{\bar{N}(t)}\right)}^{2}\;<\;\left(1-\varepsilon_{2}\right){\left(1+\varepsilon_{1}\right)}^{-2}\cdot \\ \;E\bar{N}\left(s,t\right)\cdot \\ {\;(\frac{N_{\text{popmax}}}{E\bar{N}(t)})}^{2}\cdot \end{array}\;(4.54)$$

In that estimate let us set *ε*_2_ = 0.5 and *ε*_1_ = 1. Taking into account that *E* $\bar{N}(t)$ = $\bar{F}(t)$ *N*_pop_( *t*) < *2C _F_N*_popmax_, we have that

$$\mathrm{Pr}(A_{\text{sur},s,\varepsilon_{3}}(t)|{ℬ}_{s}(t)ℬ(t))<{\bar{R}}_{3}(\varepsilon_{3},s,t),\;(4.55)$$

where

$${\bar{R}}_{3}(\varepsilon_{3},s,t)=2\;\text{exp}(-0.25\varepsilon_{3}^{2}C_{F}^{-2}c_{F}N(s,t)).\;(4.56)$$

Moreover, according to ( 4.47)

$$\mathrm{Pr}(\text{Not}\;{ℬ}_{s}(t))<R_{2}(s,t),\;(4.57)$$

and due to ( 4.17)

$$\mathrm{Pr}(\text{Not}\;ℬ(t)\nu )<R_{1}(s,t),\;(4.58)$$

where *R*_1_, *R*_2_ are defined by ( 4.37) and ( 4.38). Finally,

$$\mathrm{Pr}(A_{\text{sur},s,\varepsilon_{3}}(t))<R_{1}(s,t)+R_{2}(s,t)+{\bar{R}}_{3}(\varepsilon_{3},s,t).\;(4.59)$$

*Step 3, estimate of the number of mutants.*

Let us estimate how many individuals with genotypes *s* can mutate. The probability of mutation is *p*_mut_. Let *N*_mut_( *s*, *t*) be the number of such mutants. Let us define the event _mut, *s*_( *t*) by

$$A_{\text{mut},s}(t)=\{N_{\text{mut}}(s,t)>2p_{\text{mut}}\bar{N}(s,t)\},\;(4.60)$$

Since the random quantity *N*_mut_( *s*, *t*) is subject to the Bernoulli law, we can apply the Chernoff-Hoeffding inequality ( 4.13). Then we obtain that

$$\mathrm{Pr}(A_{\text{mut},s}(t))<\text{exp}(-D(2p_{\text{mut}}\|p_{\text{mut}})\bar{N}(s,t)),\;(4.61)$$

where, according to definition ( 4.14) of *D*( *x*|| *y*), one has

$$D(2p_{\text{mut}}\|p_{\text{mut}})=g(p_{\text{mut}})$$

and *g* is defined by ( 4.11).

Using ( 4.4) one has

$$\;\mathrm{Pr}(A_{\text{mut},s}(t))\;\leq \;\mathrm{Pr}(A_{\text{mut},s}(t)|{ℬ}_{s}(t))+\mathrm{Pr}(\text{Not}\;{ℬ}_{s}(t)).\;(4.62)$$

As a result, by Lemma 4.3 one finds

$$\mathrm{Pr}(A_{\text{mut},s}(t))\leq R_{4}+R_{5},\;(4.63)$$

where *R*_4_, *R*_5_ are defined by ( 4.40) and ( 4.41).

To prove ( 4.35), we set *ε*_3_ = *ε*_0_/2. Taking into account condition ( 4.75) for *ε*_0_ we see that if the both events Not _mut, *s*_( *t*) and Not _sur, *s*, *ε*_0_/2_( *t*) take place, then inequality ( 4.35) is fulfilled. Thus

$$\begin{array}{l} \;\mathrm{Pr}(\text{Not}\;A_{\text{mut},s}(t)\text{Not}\;A_{\text{sur},s,\varepsilon_{0}/2}(t))\;\geq \\ \;1-\mathrm{Pr}(A_{\text{mut},s}(t))-\mathrm{Pr}(A_{\text{sur},s,\varepsilon_{0}/2}(t))\;>\\ \;1-\sum_{i=1}^{5}R_{i}, \end{array}$$

where *R _i_* are defined by ( 4.37)–( 4.41).

Finally, taking into account the results of steps 1, 2 and 3 we see that estimate ( 4.35) holds with the probability Pr _t,+_. It completes the proof of ( 4.35). The second inequality ( 4.42) can be obtained in the same way.

### 4.3 Remaining part of the proof of Theorem 3.2

We use the same idea that in the proof of Theorem 3.1 but first we establish uniform bounds for the population size and other quantities involved in the proof.

*Step 1* Here we estimate the population size. Let us set

$$\varepsilon_{2}=1-\kappa >0$$

in Lemma 4.4. Let us consider the events **_ε _2__( *t*) defined by ( 4.24) in Lemma 4.4. If the events **_ε _2__( *t*) take place for all *t* ∈ [ *T*_1_, *T*_1_ + *T _c_*] and *N*_pop_(0) = *N*_popmax,_ we have that

$$2N_{\text{popmax}}>N_{\text{pop}}(t)\;(4.64)$$

$$\;>\kappa N_{\text{popmax}\;}\forall t\in [T_{1},T_{1}+T_{c}].\;(4.65)$$

Then conditions ( 3.15), ( 3.16) of Theorem 3.2 imply

$$\begin{array}{l} N\left(s,t\right)>\kappa p_{0}N_{\text{popmax}},\;N\left(\bar{s},t\right)>\kappa p_{1}N_{\text{popmax}}. \end{array}\;(4.66)$$

Those inequalities imply the following estimates for the quantities *R*_*i*_ defined by ( 4.37)–( 4.41):

$$R_{i}\left(s,t\right)>q_{i}\left(p_{0}\right),\;R_{i}\left(\bar{s},t\right)>q_{i}\left(p_{1}\right),\;(4.67)$$

where *q*_*i*_ are defined by

$$\;\begin{array}{l} q_{1}=\;\exp\left(-\left(2\ln2-1\right)c_{F}\kappa N_{\text{popmax}}\right)+\\ \;\exp\left(-c_{F}\kappa N_{\text{popmax}}\right),\;(4.68) \end{array}$$

$$\;\begin{array}{l} q_{2}\left(p\right)=\;2\exp(-\left(3/2\ln\left(3/2\right)-1/2\right)\\ \;c_{F}p\kappa N_{\text{popmax}})+\\ \;\exp(-1/2\left(1-\ln2\right)c_{F}p\\ \;\kappa N_{\text{popmax}}),\;(4.69) \end{array}$$

$$\;q_{3}\left(p\right)=\;2\exp\left(-\frac{\varepsilon_{0}^{2}}{16C_{F}^{2}}\kappa c_{F}pN_{\text{popmax}}\right),\;(4.70)$$

$$\;q_{4}\left(p\right)=\;\exp\left(-0.5U\left(p_{\text{mut}}\right)c_{F}p\kappa N_{\text{popmax}}\right),\;(4.71)$$

$$\;\begin{array}{l} q_{5}\left(p\right)=\;\exp(-\left(2\ln2-1\right)c_{F}\kappa pN_{\text{popmax}})+\\ \;\exp\left(-c_{F}p\kappa N_{\text{popmax}}\right), \end{array}\;(4.72)$$

where

U(p)=2ln⁡2p+(1−p)ln⁡((1−2p)/(1−p)),(4.73)

and

$$\;\begin{array}{l} \tilde{q}=\;\exp\left(-d\left(\tilde{\varepsilon }\right)\kappa N_{\text{popmax}}\right)+\\ \;\exp\left(-d\left(-\tilde{\varepsilon }\right)\kappa N_{\text{popmax}}\right)+\\ \;2\exp\left(-\frac{2{\left(1-\kappa \right)}^{2}N_{\text{popmax}}}{\text{2}\left(\text{1+}\tilde{\varepsilon }\right)C_{F}}\right).\;(4.74) \end{array}$$

where $\tilde{\varepsilon }$ = 1 – ( *κc*_*F*_) ^–1^, and

$$\begin{array}{l} \varepsilon_{0}=\frac{1-\exp\left(-b_{j}\delta_{j}T_{c}/2\right)}{\;1+\;\exp\left(-b_{j}\delta_{j}T_{c}/2\right)}\\ \;>4p_{mut}C_{F}.\;(4.75) \end{array}$$

For each *p* ∈ (0, 1) let us define an auxiliary function

ρ(p)=q1+q2(p)+q3(p)+q4(p)+q5(p)+q˜,(4.76)

where *q*_*i*_, $\tilde{q}$ are defined by relations ( 4.68)–( 4.72). We can find asymptotics of *ρ* under natural assumptions that *p*_mut_ → 0 and *N*_popmax_ → ∞ while all the rest parameters are fixed. Then the leading term in the right hand side of ( 4.36) is *q*_4_ and $U\left(p_{\text{mut}}\right)=\left(2\ln2-1\right)p_{\text{mut}}+O\left(p_{\text{mut}}^{2}\right).$ As a result, we have

$$\begin{array}{l} \rho \left(p\right)=\exp\left(-\left(\ln2-1/2\right)p_{\text{mut}}c_{F}p\kappa N_{\text{popmax}}\right).\\ \;(1+o\left(1\right)),p_{\text{mut}}\to 0. \end{array}\;(4.77)$$

*Step 2*. Let *Q*( *t*) is defined by ( 3.8) and, moreover, let *j* ∈ *I*_−_. We use Lemma 4.5 inductively for genotypes *s* and $\tilde{s}$. Let us set

$$\theta =\frac{1-\varepsilon_{0}}{1+\varepsilon_{0}},$$

where *ε* is defined by ( 4.75). We remark that the inequality

$$Q\left(T_{c}+t+1\right)\geq Q\left(T_{c}+t\right)\theta \exp\left(b_{j}\delta_{j}T_{c}\right)\;(4.78)$$

holds with a probability Pr _*Q*, *t*_ > 0. Let us obtain a uniform estimate of that probability. Let *ℰ*( *t*) be the event that ( 4.78) holds at the step *t*. Using ( 4.4) we have

$$\begin{array}{l} \text{Pr}\left(\text{Not}\;ℰ\left(t\right)\right)\leq \text{Pr}\left(\text{Not}\;ℰ\left(t\right)|{}_{\varepsilon_{2}}\left(t\right)\right)+\\ \;\text{Pr}\left(\text{Not}\;{}_{\varepsilon_{2}}\left(t\right)\right),\;(4.79) \end{array}$$

where, according to Lemma 4.4, the probability of the event Not **_ε _2__( *t*) is less than *η*, where *η* is defined by ( 4.25), and

$$Pr\left(Not\;ℰ\left(t\right)|{}_{\varepsilon_{2}}\left(t\right)\right)<\tilde{q}+\rho \left(p_{0}\right)+\rho \left(p_{1}\right).$$

We conclude by ( 4.5) that

$$Pr_{Q,t}>Z,\;Z=1-\tilde{q}-\rho \left(p_{0}\right)-\rho \left(p_{1}\right),\;(4.80)$$

where $\tilde{q}$ is defined by ( 4.74). This estimate is uniform in *t* *∈* [1, . . . , *T _c_*]. By ( 4.80) we obtain then that the inequality

$$Q\left(T_{c}+T_{1}\right)\geq Q\left(T_{1}\right)\bar{\theta },\;\bar{\theta }=\frac{1-\varepsilon_{0}}{1+\varepsilon_{0}}\text{exp}\left(b_{j}\delta_{j}T_{c}\right).\;(4.81)$$

is satisfied with the probability Pr _*v*_ such that

$${\text{Pr}}_{v}>Z^{T_{c}}.\;(4.82)$$

For *ε*_0_ defined by ( 4.75). one has

$$Q(T_{c}+T_{1})\geq Q(T_{1})\exp(b_{j}\delta_{j}T_{c}/2).$$

Now repeating the same arguments that in the end of the proof of Theorem 3.1, and taking into account asymptotics ( 4.77), we obtain the conclusion of Theorem 3.2.

## 5 Discussion

In this paper, we proposed a model for fitness landscape learning, which extends earlier work by 7– 9 in two ways. First, we use hybrid circuits involving two kinds of variables. The first class of variables are real valued in the interval (0, 1) and can be interpreted as relative levels of phenotypic traits, other variables are Boolean and can be interpreted as genes. Second, we use a threshold scheme of regulation, which is inspired by ideas of the paper by 20. All variables are involved in gene regulation via thresholds.

The work presented here is a major extension of a long term effort to explicitly model the effects of phenotypic buffering in evolution by considering a class of Boolean and mixed Boolean-continuous models in which the phenotype is represented explicitly and the degree of phenotypic buffering can be controlled in various ways. For example, we have demonstrated that the idea of an “evolutionary capacitor” ^42, 43^ can be implemented by explicit control of phenotypic buffering in a hub-and-spokes architecture ^23^ and that in a more general class of genetic architecture numerical simulations show that an intermediate level of buffering is optimal for evolution in a changing environment ^31^.

**Figure 4.**
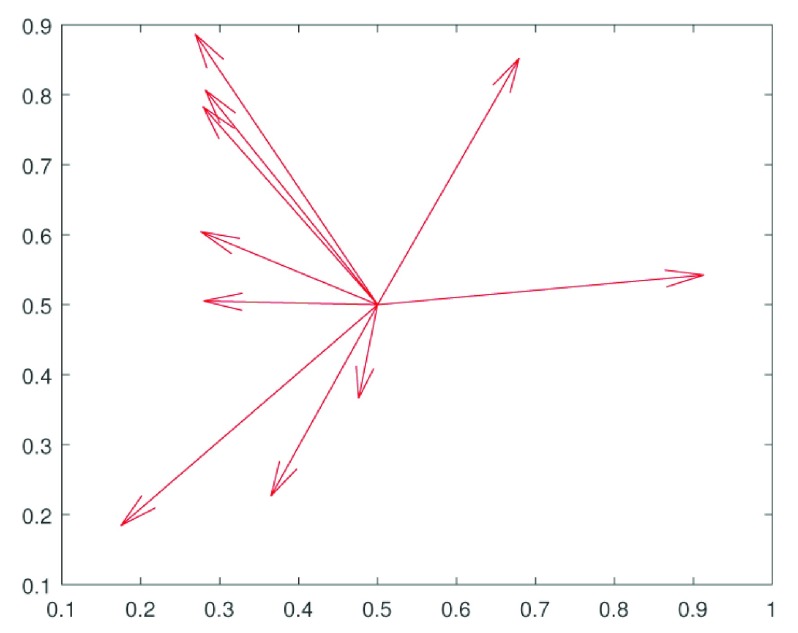
This graph illustrates Goldshmidt’s leaps. At the initial moment the trait expressions take the values *x* = 0.5, *y* = 0.5. According to Fisher’s ideas, random large mutations decrease the fitness *F* = *K _F_* exp( *W*). (Changes of *x* = *F _1_*, *y* = *f _2_*, which are induced by mutations, are shown by red vectors.) Thus such mutations produce non-viable organisms.

**Figure 5.**
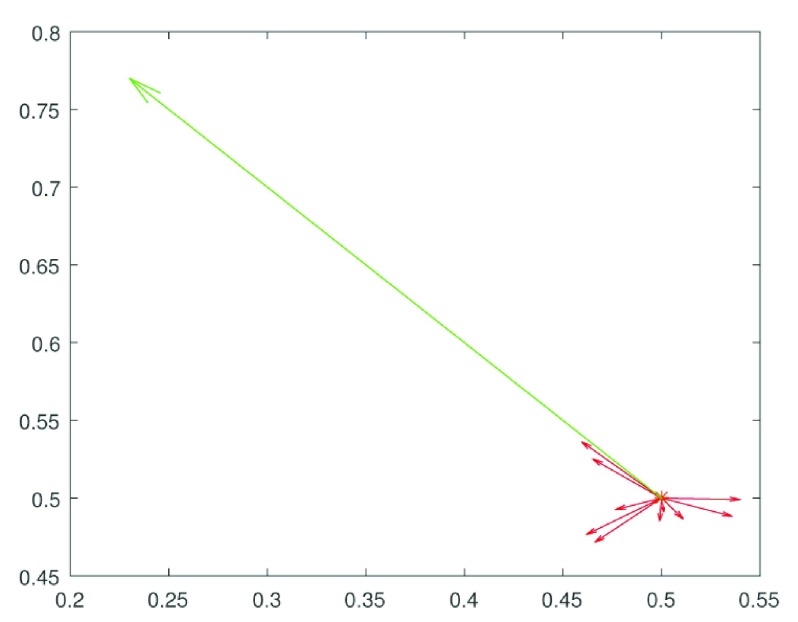
This plot illustrates the main ideas of evolution based on the fitness landscape learning. At the initial time the trait expressions take the values *x* = 0.5, *y* = 0.5. Evolutionary changes go in two stages. First we make small random mutations (shown by red vectors), which explore the fitness landscape. If such a mutation is not eliminated from the population, this means that a correct evolution direction is found, and gene regulation system makes a big leap (shown by the green vector) in the direction of that small mutation. Such a two step model can be called clever Goldshmidt leaps. Note that evolution is gradual, and the existence of clusters of almost identical genes involved in the same QTL increases the chances to create a clever Goldschmidt hopeful monster.

The results reported here are very promising, since they are consistent with the results of recent experiments by 44 and 45 on heat shock stress. The essential mechanism is that the exploration of the fitness landscape by the genetic network in such a way that future mutations are more likely to be adaptive. We have shown that, at least for some fitness landscapes, rapid evolutionary changes—perhaps instances of the “hopeful monsters” of Goldschmidt ^46^—can be created by a combination of random small mutations and epigenetic effects. The main idea is that small mutations pave the way for large epigenetic or genetic changes. The hypothetical mechanism, which we propose, can be outlined as follows (see Figure 4, Figure 5).

This network modifies the thresholds by a simple feedback mechanism. Although we are not aware of clear experimental evidence for the existence of such a mechanism, we nevertheless think that such a mechanism can be connected with regulations via enhancers ^47, 48^, where enhancer action is described by deep network models based on thermodynamics, and chemical kinetics, and those models contain threshold parameters. Alternative variants involve modifications of weights. To some extent, both mechanisms are mathematically equivalent. However, the regulation via thresholds has an important advantage: it makes phenotypes robust with respect to mutations. In this second version of the paper we included a subsection about regulation and two pictures, which show results of numerical simulations based on the “strong selection weak mutation” (SSWM) algorithm. As is the case for threshold signs, they of course should have different signs. The key question is how an individual obtains information about the fitness landscape. In our model that information is the sign of *b _j_*. If the *b _j_* is positive the corresponding threshold must be negative, otherwise, it should be negative. Actually, we think that the gene of individuals have that information! According to the main theorems proved in this paper, the fact that a mutated individual survives for a sufficient long period of time gives us that information. The very fact of the existence of the individual carries the most important information. Look at the mutations of its genotype and you will know where evolution should go!

As is described in Subsection 2.5, we assume that expression of genes involved in the expression of phenotypic traits depends on threshold parameters *h _j_*, which take three values: a large negative one, a neutral value close to zero and a large positive one. First the threshold parameter *h _j_* is small and thus the phenotypic trait is sensitive with respect to even small mutations. Those mutations play a fundamental role working as scouts exploring environments (see Figure 4). If a mutation occurred and the corresponding mutant has survived within *T _c_* ≫ 1 generations then according to Theorem 3.1 and Theorem 3.2 these events mean that that mutation increases the fitness that allows the network to estimate the correct direction of evolution. Then gene regulation detects that increase to change the threshold according to simple rules. Namely, if the trait is less expressed in that mutant with respect to wild type parent, the gene regulation system decreases the threshold up to the large negative value. On the contrary, if the trait is strongly expressed in the mutant, the gene regulation system increases the threshold up to the large positive value. This simple regulation control not only sharply reduces the number of mutations needed for adaptation, but also canalizes the phenotype since for large thresholds the trait expression level becomes insensitive with respect to mutations. We suppose that these threshold modifications can be inherited.

So, we propose the mechanism: small mutations serve as scouts finding the way for large epigenetic or genetic changes, which can be performed by gene regulatory system.

The mechanism may also explain the results of 4 on prediction of environmental changes. In fact, let us suppose that environment varies in time. The first, perhaps relatively small, variations can trigger the threshold mechanism described above. As a result, the population will be adapted to the subsequent changes in advance.

Our results show that evolution can proceed rapidly because it reduces the number of mutations required for adaptive change.

The primary limitation of our results is that the representation of the evolving genetic network is limited to the network of gene controlling phenotype, represented here by the Boolean strings *s*. Other model variables represent the coarse-grained activities of genes. One class is the terminal differentiation genes represented by *w _ij_*, and another are the genes or epigenetic factors controlling the thresholds *h* and their associated learning rules. A more careful consideration of the relationship of these moieties to observable molecular entities is an important objective of future work. At the mathematical level, the key analytical results were obtained in a simplified context that falls short of a realistic level of pleiotropy and thus of the level of NP-hard complexity exhibited by fully pleotropic forms of our model. We believe that our analytical results can be generalized, which we plan to address in future work.

## Data availability

All data underlying the results are available as part of the article and no additional source data are required.
