# Supplementary material for: Adaptation, fitness landscape learning and fast evolution
Source: F1000Res. 2019 Sep 13;8:358. Originally published 2019 Apr 1. [Version 2] doi: 10.12688/f1000research.18575.2 (PMC6798318; doi:10.12688/f1000research.18575.2)
Supplement: Peer review history

# Peer review: Reviewer response for version 2

**DOI:** 10.5256/f1000research.22586.r53938

**Published:** 2019-10-16

**Authors:** Eors Szathmary

**Affiliations:** 1 Evolutionary Systems Research Group, MTA Centre for Ecological Research, Tihany, Hungary; 2 Parmenides Center for the Conceptual Foundations of Science, Pullach, Germany; 3 Deparment of Plant Systematics, Ecology and Theoretical Biology, Eötvös University, Budapest, Hungary

This is a throught-provoking, hypothetical paper with considerable potential, duly revised.

I confirm that I have read this submission and believe that I have an appropriate level of expertise to confirm that it is of an acceptable scientific standard.

# Peer review: Reviewer response for version 2

**DOI:** 10.5256/f1000research.22586.r53939

**Published:** 2019-09-27

**Authors:** Aviv Bergman

**Affiliations:** 1 Systems & Computational Biology Department, Albert Einstein College of Medicine, New York, NY, USA

I confirm that I have read this submission and believe that I have an appropriate level of expertise to confirm that it is of an acceptable scientific standard.

# Peer review: Reviewer response for version 1

**DOI:** 10.5256/f1000research.20332.r48858

**Published:** 2019-06-13

**Authors:** Aviv Bergman, Yehonatan Sella

**Affiliations:** 1 Systems & Computational Biology Department, Albert Einstein College of Medicine, New York, NY, USA

In the current work the authors study a population genetics model in which fitness is a linear function of a set of phenotypic traits, and where the genotype-to-phenotype map is given by a linear transformation composed with sigmoidal functions. Despite the seeming simplicity of the fitness function, the authors make the case that optimizing fitness is a hard, NP-complete, problem. Under this model, they study the extent to which the fitness landscape (that is, the question of which phenotypic traits contribute positively to fitness, and which contribute negatively) can be inferred from the distribution of these traits in the population after being subject to evolution for a moderate span of time. They then connect this question with that of whether learning algorithms (potentially epigenetic in nature) can help optimize and speed up evolution by learning the fitness landscape.

 Toward the goal of inferring the fitness landscape, the authors prove two theorems. Theorem 3.1 concerns a simplified model of an infinite population with no mutations, while Theorem 3.2 concerns a more complex model of a finite population with mutation, stochastic number of descendants and a culling process. In either case, the result is that under certain assumptions, if a mutant genotype is present in the population with high enough frequency after a long enough period of elapsed time, then we can confidently infer that any phenotypic trait differential between the wildtype and the mutant is associated with a higher fitness.

 While Theorem 3.1 ignores mutation, even Theorem 3.2 seems at odds with mutation-selection balance. Even a mutant with lower fitness will be present in the population at a frequency that is on the order of the rate of mutation, while the theorem seems to claim that the frequency of such a mutant will be exponentially small with high probability. The authors should resolve this apparent discrepancy. In addition, the main ideas of the proof would be more clearly communicated if the authors would include a treatment of the intermediate model of an infinite population with mutation.

 As for the discussion of the way learning can speed up the evolutionary process, this part of the paper remains unclear and underdeveloped. The authors discuss a two-step evolutionary process in which the first step consists of small mutations in order to explore the fitness landscape, and the next step involves changing the thresholds involved in the genotype-to-phenotype map in a way that promotes phenotypic traits associated with higher fitness. While this idea is interesting and worth exploring, a few issues arise. A conceptual issue remaining to be addressed is whether the threshold h _i _is part of the genotype and what mechanism are needed to alter its value. According to the authors, the h _i_’s can be modified genetically or epigenetically. If epigenetically, it is not apparent what are the environmental cues that will lead to such learning let alone the actual mechanism of modifying them.  If genetically, it is similarly unclear in what way the learning of the fitness landscape is being stored, if at all, in the genotype, and what is the connection to the Theorems of chapter 3. The theorems in chapter 3 rely on observing the frequency of a genotype in the population, but such information is not stored in individual genotypes.  Additionally, if the thresholds are understood to be variable and subject to selection, then the fitness-maximization problem in fact becomes easy (in contrast to the prior analysis of it as NP-complete), unless we impose restrictions on the range of the threshold. Indeed, one can set the thresholds at positive or negative infinity depending on whether the corresponding trait is positive or negative in order to effectively keep the trait on or off regardless of genotype. 

 Finally, as this manuscript addresses the relation between learning and the rate of evolution, it would benefit from including a reference to one of the most relevant and intuitive articles written in the late 80’s by Geoffrey E. Hinton & Steven J. Nowlan, “How Learning Can Guide Evolution” *Complex Systems*, 1, 495-502 ^1^. In it, Hinton and Nowlan showed how learning alters the shape of the fitness landscape and thereby provides easier evolutionary paths towards sets of co-adapted alleles. Hinton and Nowlan demonstrated that this effect allows learning individuals to evolve faster than non-learners. Though the learning model presented by Hinton and Nowlan operates at “somatic” timescale, the analogy to mutations at the evolutionary timescale can be drawn.

 To conclude, though it can be improved by the above suggestions, this article touches upon very interesting and important issues in the field of evolutionary biology which are still only lightly investigated, and highlight what might be a fruitful path towards better understanding.

We confirm that we have read this submission and believe that we have an appropriate level of expertise to confirm that it is of an acceptable scientific standard, however we have significant reservations, as outlined above.

## References

[1] : How Learning Can Guide Evolution. *Complex Systems*.1987;1:

# Peer review: Reviewer response for version 1

**DOI:** 10.5256/f1000research.20332.r46602

**Published:** 2019-04-23

**Authors:** Eors Szathmary

**Affiliations:** 1 Evolutionary Systems Research Group, MTA Centre for Ecological Research, Tihany, Hungary; 2 Parmenides Center for the Conceptual Foundations of Science, Pullach, Germany; 3 Deparment of Plant Systematics, Ecology and Theoretical Biology, Eötvös University, Budapest, Hungary

This is a new paper in a series of exciting papers about phenotypic evolution, based on models of genes genetic regulatory networks, and phenotypes.

 There are many technical details in the paper that most biologists will find difficult to follow – I would put at least some of these items into an Appendix. Also, I am wondering how many (even theoretical) biologists would readily grasp the meaning of the K-SAT problem as defined. A more accessible formulation (in addition to the strictly technical one) could do a lot for easier understanding (one of the papers by the authors contains a didactive figure, for example).

 I consider this paper a serious attempt at broadening our views of the role of learning dynamics in evolution, but I think the message could be made a lot clearer. The punch line in a way is in the Discussion that I find both intriguing and baffling.

 First, as it is portrayed in this paper, the effect of genes is filtered through a genetic regulatory network. It seems that, just like in Ref 40 ^1^, that this filter itself does not evolve, or at least the dynamics is not given. In other words, how do the weights in the network evolve? Note that in the Watson-Wagner paper in *Evolution* ^2^ it was the evolution of the weights that was related to Hebbian dynamics. So, how do we stand on this?

 Second, in this paper the genetic control of the thresholds in also kept implicit, but the evolution of threshold values does play an important role in the Goldschmidtian argument. Is the change in thresholds genetic or epigenetic? If the latter, then it ought to be part of the regulatory network. I guess this is what the statement “gene regulation detects that increase to change the threshold according to simple rules”. How these rules would be implemented in mechanistic terms remains obscure. And how would they arise in evolution? The simple rule implies feedback from expression levels to threshold values.

 Third, do I understand it correctly that here, in contrast to Ref 40 ^1^, thresholds can be negative? But a large negative threshold value would mean that expression levels will increase, since negative times negative is positive (Eq. 2.4). If the statement “take three values: a large negative one, a neutral value close to zero and a large positive one” instead refers to gene expression levels rather than threshold values (the latter would in this case be nonnegative) then I do not understand the thought example. Increasing the threshold would reduce the expression level that was selected to go up in the first place!

 These points seem to me crucial, so their clarification is badly needed. I would appreciate a few numerical examples along with at least hints to answers to the more conceptual questions on the dynamics.

 Also, according to the cited Nagylaki dynamics ^3^ the population evolves almost as if it were in linkage equilibrium – which cannot hold for the asexual population considered by the authors.

 There are also some minor issues in the paper: in Eq. 2.4 “s” should be replaced by a sigma, and there are also examples where plurals and singulars in the same sentence do not match.

I confirm that I have read this submission and believe that I have an appropriate level of expertise to confirm that it is of an acceptable scientific standard, however I have significant reservations, as outlined above.

## References

[1] : The relationship between robustness and evolution. *bioRxiv*.2018; 10.1101/268862 10.1101/268862

[2] : The evolution of phenotypic correlations and. *Evolution*.2014;68(4) : 10.1111/evo.12337 1124-38 10.1111/evo.12337 24351058PMC4131988

[3] : The evolution of multilocus systems under weak selection. *Genetics*.1993;134(2) :627-47 832549210.1093/genetics/134.2.627PMC1205503
